# Vaginal stump rupture due to sexual activity leading to small intestine vaginal hernia and intestinal necrosis in a woman after cervical cancer surgery: A case report

**DOI:** 10.1097/MD.0000000000041788

**Published:** 2025-04-18

**Authors:** Shenyi Lu, Huadi Yang, Peiyu Mao, Guiping Chen

**Affiliations:** a Department of Gynecology and Obstetrics, The First Affiliated Hospital of Zhejiang Chinese Medical University (Zhejiang Provincial Hospital of Chinese Medicine), Hangzhou, Zhejiang Province, China.

**Keywords:** cervical cancer, intestinal necrosis, sexual activity, vaginal stump rupture

## Abstract

**Rationale:**

Radical hysterectomy is the standard surgical procedure for early-stage cervical cancer, and postoperative adjuvant chemoradiotherapy is administered based on pathological high-risk and intermediate-risk factors. After treatment of cervical cancer, all focus is placed on postoperative recurrence, metastasis and menopausal symptoms, and almost no one pays attention to the recovery of vaginal elasticity and sexual activity of reproductive-age women. Due to frequent surgery and radiation therapy for cervical cancer patients, the length and elasticity of the vagina are reduced. Cervical cancer patients need to be aware of issues related to sexual activity, such as vaginal shortening or shrinking, the impact of cancer on sexuality, and when to restore sexual activity after surgery. Patients who receive this information can better cope with these changes. We report a clinical case with the aim of sharing our experiences and calling for an emphasis on the recovery of vaginal stump and vaginal elasticity and sexual activity guidance after radical hysterectomy.

**Patient concerns:**

A 48-year-old woman with cervical cancer who underwent radical hysterectomy 6 years ago was admitted to the gastrointestinal surgery ward urgently, having shown severe upper abdominal pain accompanied 6 hours after sex activity.

**Diagnoses:**

Small bowel vaginal hernia with intestinal necrosis was diagnosed.

**Interventions:**

Urgent surgery was performed to resect the necrotic small intestine and repair the vaginal stump and pelvic floor.

**Outcomes:**

After surgery, she recovered well and is currently under regular follow-up for cervical cancer.

**Lessons:**

It is crucial to regularly guide and follow-up on patients’ sexual behavior during the follow-up period after cervical cancer surgery. Gynecologists should fully inform the time for restoring sexual relations after cervical cancer surgery. Nursing staff should be guided to avoid premature and excessive sexual activity to the vaginal cuff to prevent rupture and other potential complications. Effective medical intervention should be implemented as early as possible for patients with vaginal sac rupture after cervical cancer surgery. Current research on sexual activity after cervical cancer surgery has many limitations and more detailed studies are needed. A specific questionnaire to assess sexual activity and a clinical study to determine the appropriate medication to restore vaginal elasticity are expected.

## 1. Introduction

Cervical cancer is the fourth most common cancer amongst women worldwide.^[1]^ Advances in cervical cancer screening and treatment have resulted in high cure rates in developed countries for early-stage disease.^[2]^ Surgery plays an important role in the management of early-stage cervical cancer, with the radical hysterectomy being the standard surgical procedure.^[3]^ Postoperative adjuvant chemoradiotherapy is administered based on pathological high and intermediate-risk factors, and definitive concurrent chemoradiotherapy can be employed at all stages, while comprehensive treatment is performed for advanced and recurrent patients.^[4]^

All focus is placed on postoperative recurrence, metastasis and menopausal symptoms, and almost no one pays attention to the sexual activity of reproductive age women after radical hysterectomy. This case describes a woman of postoperative cervical cancer suffered from small intestine vaginal hernia and intestinal necrosis due to rupture vaginal stump after sexual activity, with the aim of calling for emphasis on the recovery of vaginal stump and vaginal elasticity and sexual activity guidance after radical hysterectomy.

## 2. Case presentation

**Figure 1.**
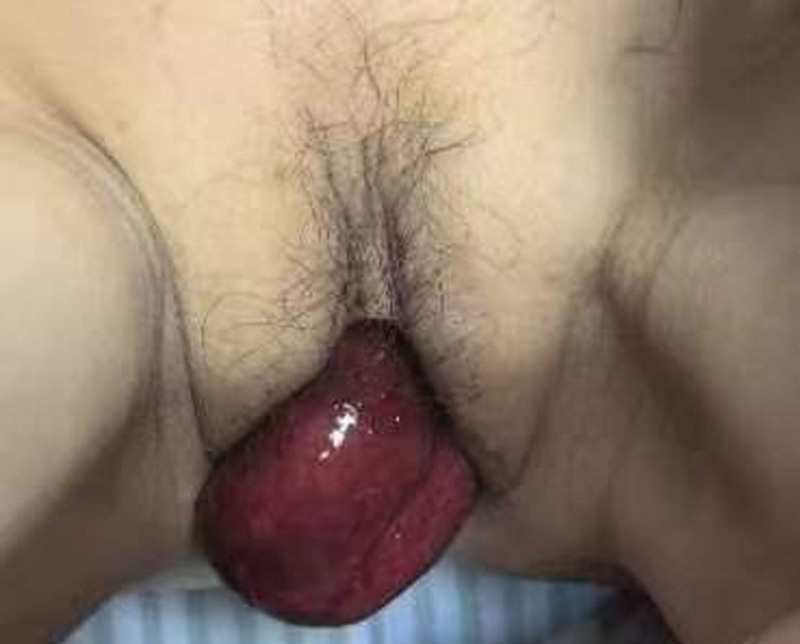
Preoperative physical examination revealed small intestine hernia from the vagina.

A 48-year-old woman was admitted to the gastrointestinal surgery ward urgently, having shown severe upper abdominal pain accompanied by nausea and vomiting 6 hours after sex activity. Physical examination revealed approximately 10 cm of intestine, which appeared purplish-black, cold touch and little peristalsis, prolapsing through the vagina (Fig. 1). Medical history guided us that she received radical hysterectomy because of early-stage cervical cancer 6 years ago, and experienced a recurrence during routine follow-up. After discovering the recurrence of cervical cancer, the patient received synchronous radiotherapy and chemotherapy.

**Figure 2.**
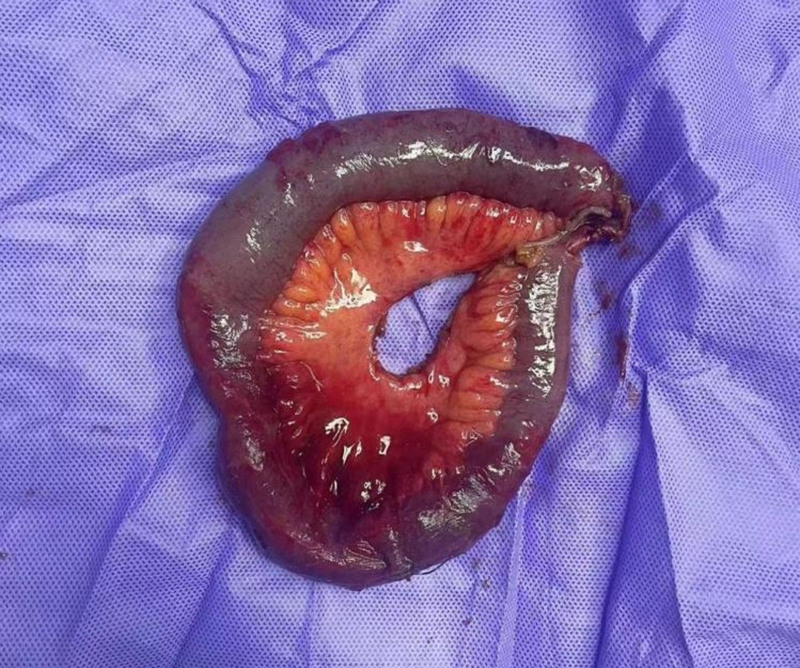
The prolapsed intestinal tissues that had entered the vagina through the ruptured vaginal cuff was resected.

**Figure 3.**
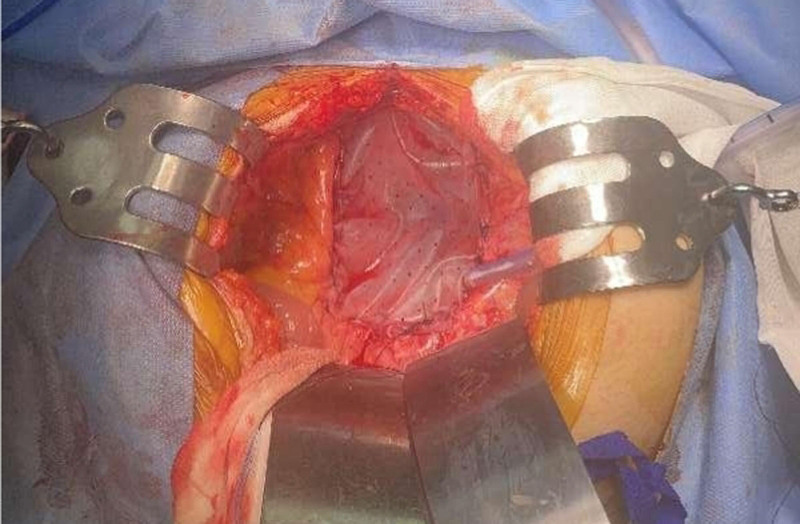
A biobased membrane patch was used to repair the pelvic floor.

Small bowel vaginal hernia with intestinal necrosis was diagnosed and urgent surgery was performed. During the surgery, we carefully denuded the necrotic small intestine at both proximal and distal ends, excised approximately 20 cm of the necrotic small intestine (Fig. 2), and the proximal and distal small intestine were lateral anastomosed. The vaginal laceration was closed from the perineum, leaving a residual vaginal of approximately 2 to 3 cm. A biologic basement membrane patch was used to repair the pelvic floor (Fig. 3).

After surgery, she was transferred back to the gastrointestinal surgery ward and received anti infection treatment. Histopathological evaluation revealed necrotic small intestine. We reported the case at 6 months following surgery, and the patient had recovered very well with a repaired vaginal stump. Whole abdominal computed tomography showed nothing abnormal. She is currently under regular follow-up of cervical cancer.

## 3. Discussion

Globally, the average age at diagnosis of cervical cancer was 53 years, ranging from 44 years (Vanuatu) to 68 years (Singapore).^[5]^ Health care workers need to be aware that women between these ages still have sexual activity. Though a study revealed loss of the desire to engage in sexual activity in some patients with cervical cancer after surgery,^[6]^ their healthy male companions’s sexual desire in this age group will not be compromised.

The life quality of cervical cancer patients is severely compromised due to the vaginal stump contracture after surgery combined with radiotherapy.^[7]^ In this case, the length of vagina was shorter after radical hysterectomy, and the vaginal stump was contracted after radiotherapy. The loss of elasticity and resilience of vaginal cuff led to the rupture under intense strain. The gap between the vaginal cuff allowed small bowel tissue to protrude through the rupture site into the vagina, causing small bowel incarceration and intestinal necrosis. This is also related to the lack of sexual guidance for both spouses. Counseling about these issues is important and should be addressed. Currently, there is a lack of literature reports on this topic.

In China, women usually do not actively seek advice on sexual life due to their conservative attitudes towards sexuality. It is, therefore, advisable for gynecologists to address their patients’ sexuality routinely during the follow-up period after cervical cancer surgery. After treatment of cervical cancer, medications for restoring vaginal elasticity should be administered. Time to resume sexual relations after surgery and guidance of care taken to avoid excessive stimulation and trauma to the vaginal cuff to prevent rupture and potential complication during sexual activity should be informed. For patients who experience vaginal cuff rupture, early medical intervention is essential to prevent further complications.

Sexuality has an important impact on people’s physical and mental health, but current research on the sexual activity of cervical cancer after surgery has many limitations. The current research on the universal effectiveness of sexual guidance after cervical cancer surgery is limited by the lack of clinical observation method design, and more detailed studies are needed. A specific questionnaire to assess the sexual activity and a clinical study to determine the appropriate medication to restore vaginal elasticity are expected.

## Author contributions

**Data curation:** Huadi Yang, Peiyu Mao.

**Writing – original draft:** Shenyi Lu, Guiping Chen.

**Writing – review & editing:** Shenyi Lu, Huadi Yang, Guiping Chen.
